# Age category-related differences in pacing during biathlon sprint competition

**DOI:** 10.3389/fspor.2026.1875719

**Published:** 2026-07-27

**Authors:** Marko S. Laaksonen

**Affiliations:** Swedish Winter Sports Research Centre, Department of Health Sciences, Mid Sweden University, Östersund, Sweden

**Keywords:** athlete development, biathlete, experience, maturation, pacing strategy, performance, skiing

## Abstract

**Introduction:**

The present study aimed to compare pacing patterns among the best international level senior, junior, and youth biathletes in both sexes during a biathlon sprint competition, while also accounting for overall performance level.

**Methods:**

Skiing speed and shooting performance were analysed from international biathlon sprint competitions held at the same venue, including senior, junior, and youth biathletes ranked 1–30 and 31–60. Relative skiing speed (SS_REL_, %) was calculated for each lap (1–3) and for three sections (A–C) within laps based on each biathlete's mean skiing speed across all laps and sections. Differences in SS_REL_ were compared between age categories and performance groups across laps and sections using repeated-measures ANOVA Additional repeated-measures ANOVA adjusting for penalty time from the preceding shooting bout during laps 2 and 3 was also performed.

**Results:**

During lap 1, senior women and men demonstrated slower SS_REL_ than junior and youth biathletes (*p* < 0.05). In contrast, during laps 2 and 3 they showed faster SS_REL_ than both younger groups (*p* < 0.05). These differences were mainly related to variations in SS_REL_ in sections A and C (*p* < 0.05). Higher-performing biathletes of both sexes showed slower SS_REL_ during lap 1 (*p* < 0.05), whereas during lap 3 they had faster SS_REL_ (only men) than lower-performing biathletes (*p* < 0.05). Including penalty time after prone and standing shooting as a covariate revealed significant effects on subsequent sectional SS_REL_ (*p* < 0.05) but did not alter pairwise group comparisons.

**Conclusion:**

The present results suggest that senior biathletes exhibit more even pacing than junior and youth biathletes across laps and sections. A similar pacing pattern was also observed among higher-performing male biathletes. Although shooting performance was associated with subsequent pacing, its influence remains unclear and warrants further investigation. Monitoring pacing during training and competition may therefore help biathletes to develop more effective pacing strategies and improve skiing performance in biathlon.

## 1 Introduction

Performance in biathlon, which combines cross-country skiing and rifle marksmanship, is determined by three main components: skiing speed, shooting accuracy, and shooting speed (1). The relative importance of these components depends on the competition format. For example, in the individual competition, where each missed shot results in a 1-minute time penalty, shooting accuracy has a greater influence on final ranking (2, 3). In contrast, in sprint competitions, skiing speed typically explains more than 50% of the variance in overall performance (2, 4).

Biathlon sprint competitions cover a total skiing distance of 7.5 km for women and 10 km for men in the senior and junior categories, while the corresponding distances in youth categories are 6 km for women and 7.5 km for men (5). Biathletes start in a randomized order at 30-second intervals and complete three skiing laps, interspersed with one prone and one standing shooting bout (5). Similar to cross-country skiing, biathlon skiing takes place in varied terrain including uphill, downhill, and flat sections, resulting in substantial fluctuations in exercise intensity and speed (6–8). However, unlike cross-country skiing, skiing in biathlon is interrupted by shooting bouts, giving the sport its unique interval-like physiological nature.

Pacing can be conceptualized as the behavioural manifestation of a dynamic and continuous decision-making process in which perceptions and actions are inherently coupled (9). It refers to the strategic regulation and distribution of an athlete's energetic resources throughout a competition and represents a key determinant of performance outcomes (10, 11). In biathlon sprint, a J-shaped pacing profile has commonly been observed (2, 4, 12, 13), characterized by a fast first lap prior to prone shooting, followed by a slower second lap before standing shooting, and a faster final lap. However, given the interval-like nature of biathlon and the individual start format, biathletes must continuously optimize skiing speed while simultaneously preserving the fine motor control and postural stability required for accurate shooting (1, 14–16). This requires a dynamic, context-dependent process of real-time decision-making influenced by physiological state, course topography, and shooting proficiency (15), all under considerable physiological and cognitive load (17). Thus, as exercise intensity has been shown to affect the shooting accuracy (18, 19), the biathletes need to control the exercise intensity prior to shooting. Indeed, recent findings by Losnegard et al. suggest that biathletes intentionally reduce exercise intensity during the final 60 s preceding shooting, contributing to the characteristic pacing pattern observed in the sport (15).

Research in cross-country skiing indicates that higher-performing skiers are better able to maintain their velocity throughout a race than lower-performing skiers (20, 21). Similar tendencies have also been reported in biathlon (2–4), although shooting interruptions may substantially influence pacing in this sport. In addition, age has been shown to influence pacing strategies in several endurance sports, including speed skating (22), running (23, 24), and cross-country skiing (25). These studies generally indicate that younger or adolescent athletes adopt a less conservative pacing strategies compared with older, more experienced athletes. However, no previous study has examined whether age influences pacing patterns in biathlon competitions.

Given the unique interval-like nature of biathlon, which combines high-intensity cross-country skiing with intermittent shooting bouts, the present study aimed to compare pacing patterns in an ecological setting among the best international level biathletes in senior, junior, and youth categories during an international biathlon sprint competition while also accounting for overall performance level. In addition, the present study investigated whether potential differences in pacing patterns between age categories are related to specific sections within each skiing lap. Based on previous literature, it was hypothesized that senior biathletes, as well as those with higher performance levels, would demonstrate more even pacing strategies than their younger and lower-performing counterparts.

## 2 Methods

### 2.1 Procedure

The data were obtained from the International Biathlon Union's (IBU) *Datacenter*, an a publicly accessible database available at https://www.biathlonworld.com (26). The IBU granted permission to use these data for scientific purposes. Since the purpose of this study was to compare different age categories, and the IBU does not organize events in which all three categories compete simultaneously, data were selected from the IBU Youth/Junior World Championships and the IBU World Cup. The primary inclusion criterion was that events were held at the same venue and on identical skiing courses. A secondary criterion was that events had to take place within the previous three years (seasons 2023/2024–2025/2026), considering the fluorine ban that came into effect on July 23, 2023, as well as potential developments in equipment. Accordingly, sprint events from the IBU Youth/Junior World Championships (March 2025) and the IBU World Cup (November 2025), both held in Östersund, Sweden, were included in the final dataset.

Data were collected for biathletes ranked 1–60 in senior, junior, and youth categories for both sexes. The best 60 biathletes were chosen because these biathletes are eligible to start in the subsequent biathlon pursuit competition and are therefore likely to have exerted full effort during the analysed sprint competition. Within each age category, these 60 biathletes were subsequently divided into two equally sized performance groups (PG30, ranks 1–30 and PG60, ranks 31–60). This grouping enabled comparisons between performance levels while maintaining adequate sample size and reasonable statistical power. Accordingly, the six groups within both sexes were analysed (Table 1).

**Table 1:** Age categories, performance groups, and participant ages (median [IQR]) for women and men.

Women	Rank 1–30	Age	Rank 31–60	Age
Senior women (SW)	SW30	26 [7]	SW60	28 [6]
Junior women (JW)	JW30	20 [1]	JW60	20 [2]
Youth women (YW)	YW30	18 [1]	YW60	18 [1]
	Performance group 30 (PG30)		Performance group 60 (PG60)	

Men	Rank 1–30	Age	Rank 31–60	Age
Senior men (SM)	SM30	28 [8]	SM60	27 [8]
Junior men (JM)	JM30	21 [1]	JM60	20 [2]
Youth men (YM)	YM30	18 [1]	YM60	18 [1]
	Performance group 30 (PG30)		Performance group 60 (PG60)	

The dataset included skiing times (laps 1, 2, and 3, and total skiing time), intermediate times (three per lap), as well as shooting performance in terms of number of missed targets (MT) and penalty time (PT) for both prone and standing shooting. Penalty time (PT) was defined as the time spent skiing from the end of the shooting range to the first timing point marking the start of the subsequent skiing lap (located 40 m from the end of the shooting range). It also includes the time spent skiing penalty loop(s) (150 m each) in cases the biathlete had missed target(s). Time spent on the shooting range was excluded.

### 2.2 Weather conditions, course profiles and sections

**Figure 1:**
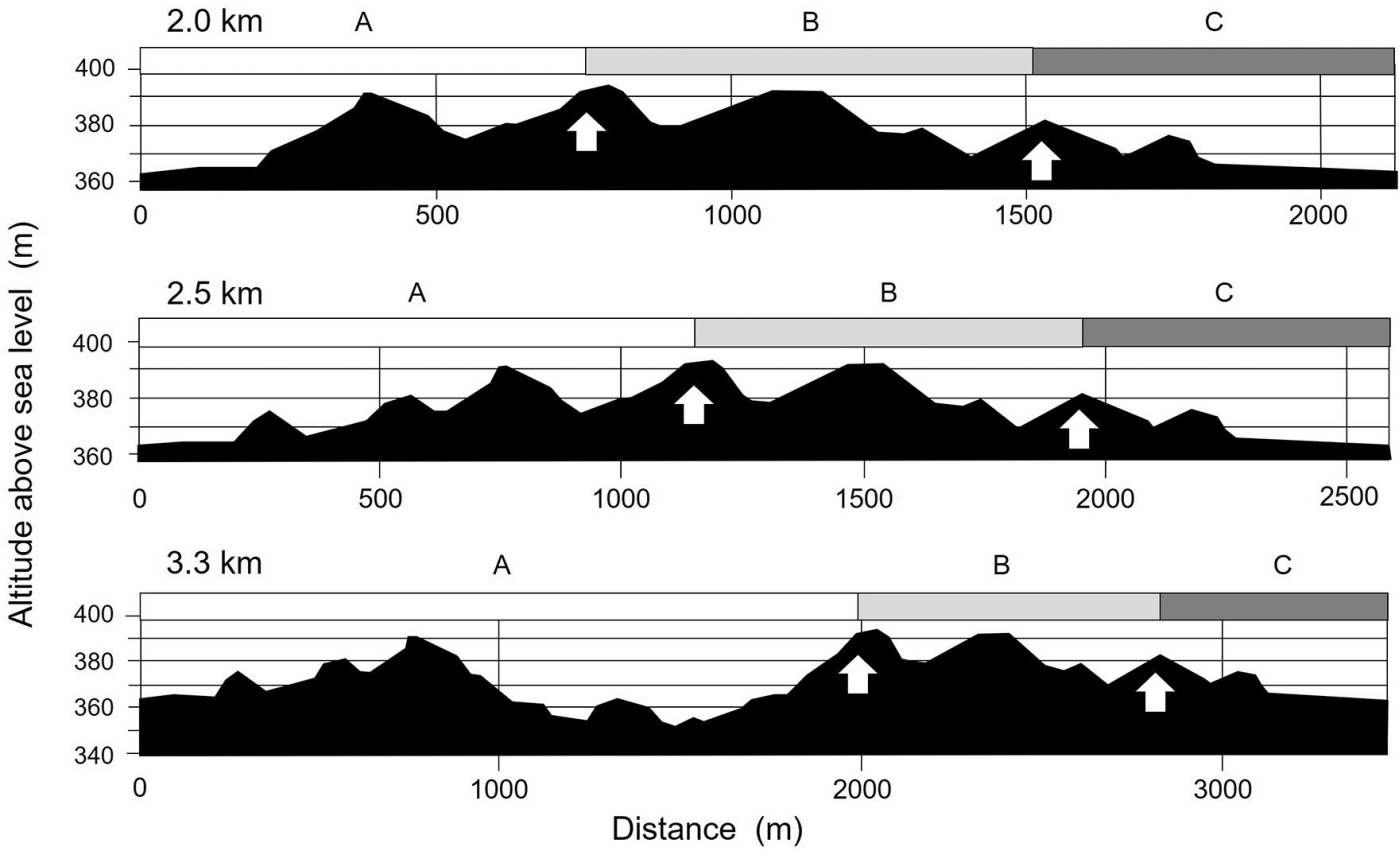
The schematic course profiles of the three courses skied during the sprint competitions (2.0 km, 2.5 km and 3.3 km) and the placement of intermediate timing points (white arrows) within each course. The white, light grey and dark grey boxes illustrate sections A, B and C, respectively.

Weather conditions and competition details are presented in Table 2. The course lengths for each category followed the International Biathlon Union's competition rules: 2.0 km for youth women (YW), 2.5 km for senior women (SW), junior women (JW), and youth men (YM), and 3.3 km for senior men (SM) and junior men (JM), with an allowed deviation of −2 to +5% of the course length. Each course was skied three times. Each lap was divided into three sections (A, B, and C; Figure 1) based on the locations of the intermediate timing points, which were positioned identically across all competitions. Section A extended from the start/first timing point after shooting range to the first intermediate timing point on the course, section B from the first to the second intermediate timing point, and section C from the second intermediate timing point to the start of the shooting range/finish. The length of each section was provided by the event organizer and based on course homologation data (Table 3). The shooting range and penalty loop were excluded from the analysis of skiing speed; time spent on possible penalty loop(s) was only counted as penalty time (see *Procedure* above).

**Table 2:** Competition dates, start times, and weather conditions across the six age categories.

Competition parameters	SW	JW	YW	SM	JM	YM
Date	2025-12-05	2025-03-02	2025-03-01	2025-12-06	2025-03-02	2025-03-01
Time	4.00 pm	0.45 pm	12.00 am	4.30 pm	4.05 pm	3.20 pm
Air Temperature (°C)	−0.7	1.2	3.7	0.0	1.0	3.5
Snow Temperature (°C)	−0.7	−0.1	0.0	−0.4	−0.2	−0.2
Humidity (%)	88	87	63	88	88	60
Wind speed (m·s−1)	0.6	1.4	2.0	1.6	1.4	1.2
Wind direction	East	West	Southwest	East	Southwest	Southwest
Weather	Overcast	Light snow/ Overcast	Overcast	Light Snow	Light snow	Overcast
Snow condition	Hard packed variable	Hard packed variable	Hard packed variable	Hard packed variable	Hard packed variable	Hard packed variable

SW, senior women; JW, junior women; YW, youth women; SM, senior men; JM, junior men; YM, youth men.

**Table 3:** Lap and section lengths, and elevation metrics (all values in meters) across age categories.

Course parameters	SM	JM	SW	JW, YM	YW
Lap 1	3,387	3,387	2,530	2,530	2,147
Lap 2	3,307	3,307	2,448	2,448	2,065
Lap 3	3,204	3,179	2,345	2,320	1,937
Total length	9,898	9,873	7,323	7,298	6,149
A – Lap 1	1,977	1,977	1,120	1,120	737
B – Lap 1	820	820	820	820	820
C – Lap 1	590	590	590	590	590
A – Lap 2	1,897	1,897	1,038	1,038	655
B – Lap 2	820	820	820	820	820
C – Lap 2	590	590	590	590	590
A – Lap 3	1,897	1,897	1,038	1,038	655
B – Lap 3	820	820	820	820	820
C – Lap 3	487	462	487	462	462
Total length	9,898	9,873	7,327	7,298	6,149
HD	42	42	29	29	29
MC	42	42	18	18	27
TC	369	369	273	273	231

SM, senior men; JM, junior men; SW, senior women; JW, junior women; YM, youth men; YW, youth women.

A, B and C refer to different sections within each lap. HD, height difference; MC maximal climb; TC total climb.

### 2.3 Calculations

First, individual absolute skiing speeds were calculated for the entire competition (all three laps combined), for each individual lap, and for each section within the laps by dividing the length of each skiing segment by the corresponding skiing time. To enable comparisons of pacing between groups, absolute skiing speed was subsequently expressed as relative skiing speed (SS_REL_, %). For each lap, SS_REL_ was calculated by dividing the absolute skiing speed by the biathlete's mean absolute skiing speed for the entire competition and multiplying by 100. Similarly, sectional speeds (A, B, and C) were converted to SS_REL_ by dividing the speed in each section by the biathlete's mean skiing speed for the corresponding section across all three laps and multiplying by 100. Shooting performance in the prone and standing positions was defined as the number of missed targets (MT_PRONE_ and MT_STAND_) and the associated penalty time (PT_PRONE_ and PT_STAND_), respectively.

### 2.4 Statistical analysis

Data were first assessed for normality using visual inspection of Q–Q plots and histograms, as well as the Shapiro–Wilk test. As the shooting-related variables were not normally distributed, differences between performance groups in MT_PRONE_, MT_STAND_, PT_PRONE_, and PT_STAND_ were analysed using a non-parametric Kruskal–Wallis ANOVA with Dwass-Steel-Critchlow-Fligner pairwise comparisons and calculations of epsilon squared effect sizes (*ε*^2^). For the normally distributed SS_REL_, a repeated-measures ANOVA (3 × 3 × 2) was performed to compare three laps (L: lap 1, 2 and 3) or sections within laps (S: section A, B and C), three age categories (AC: senior, junior and youth), and two performance groups (PG: rank 1–30, PG30 and rank 31–60, PG60). Interaction effects (L × AC, L × PG and L × AC × PG; S × AC, S × PG and S × AC × PG) were also examined. For laps 2 (after prone shooting) and 3 (after standing shooting), additional repeated-measures ANOVAs were conducted with PT_PRONE_ or PT_STAND_ included as covariates, respectively, to account for the potential influence of shooting performance on SS_REL_ in the subsequent skiing sections. All comparisons were conducted separately within each sex.

The assumption of sphericity was tested using Mauchly's test, and when violated, the degrees of freedom were corrected using the Greenhouse–Geisser adjustment. Partial eta-squared effect sizes (*η*^2^_p_) were reported for the repeated-measures ANOVA. When significant main or interaction effects were observed, Bonferroni-adjusted *post hoc* tests were performed. Due to the large number of potential pairwise comparisons in the presence of interaction effects, only comparisons within the same lap or section were considered. For simplicity, a significance level of *p* < 0.05 was used for all pairwise comparisons.

Effect sizes (*η*^2^_p_ and *ε*^2^) were interpreted as small (≥ 0.01), medium (≥ 0.06), and large (≥ 0.14) (27). All statistical analyses were performed using *jamovi* (version 2.6, The jamovi Project, 2024), with the significance level set *a priori* at α < 0.05. The data are presented as mean ± 95% confidence interval (CI) or median [interquartile range, IQR], specified separately at each instance.

## 3 Results

### 3.1 Between and within laps pacing for women

**Figure 2:**
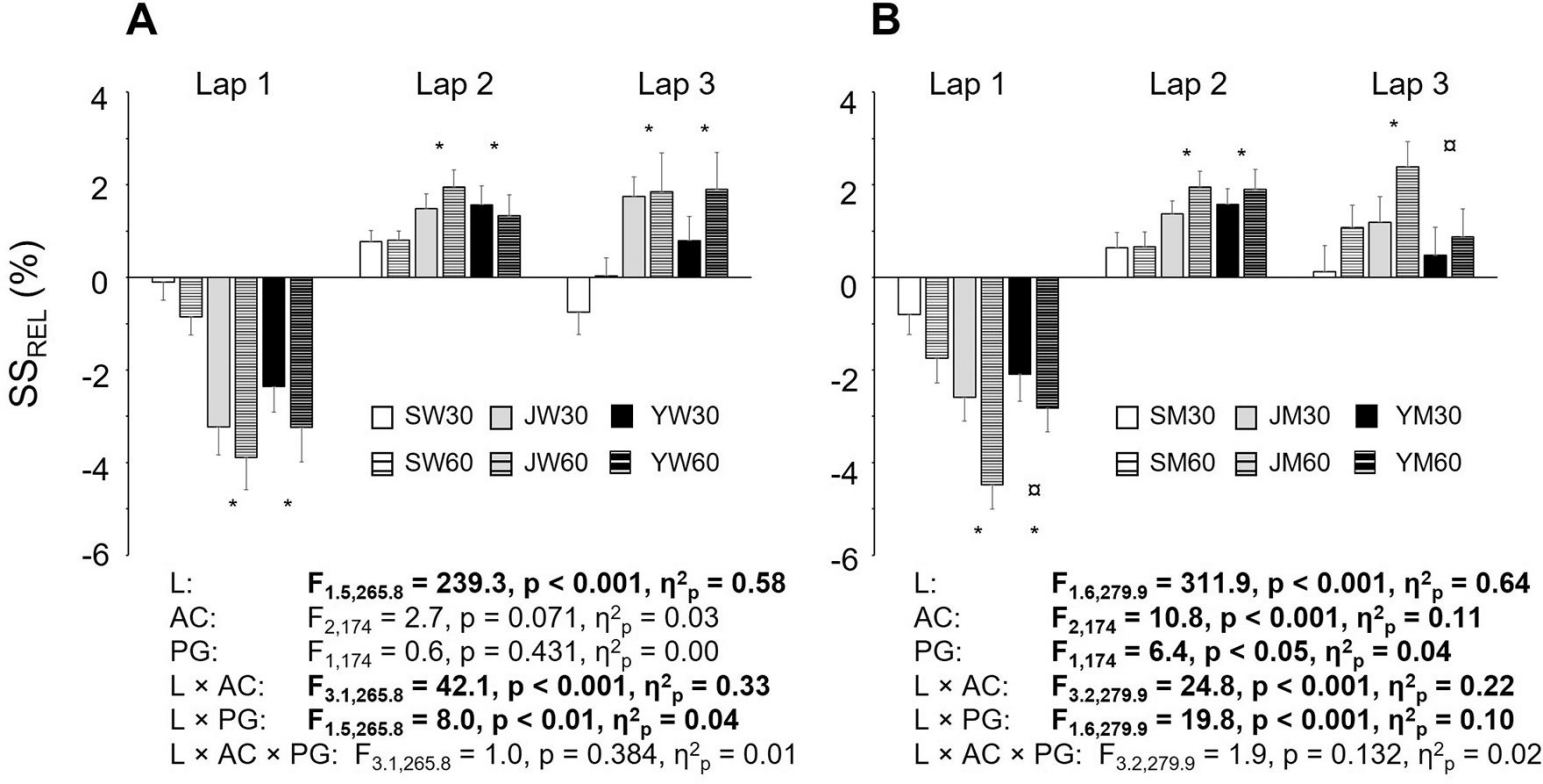
The relative skiing speed (SS_REL_, %) during the laps 1, 2 and 3 for women **(A)** and men **(B)** with the results of repeated-measures ANOVA. L, laps; AC, age categories; PG, performance groups; SW30, senior women ranked 1–30; SW60, senior women ranked 31–60; JW30, junior women ranked 1–30; JW60, junior women ranked 31–60; YW30, youth women ranked 1–30; YW60, youth women ranked 31–60; SM30, senior men ranked 1–30; SM60, senior men ranked 31–60; JM30, junior men ranked 1–30; JM60, junior men ranked 31–60; YM30, youth men ranked 1–30; YM60, youth men ranked 31–60. * *p* < 0.05 vs. SW/SM and ¤ *p* < 0.05 vs. JM based on significant L × AC interaction effect.

The results of repeated-measures ANOVA including F-values, significance levels, and effect sizes for SS_REL_ for women during all three laps are presented in Figure 2. SS_REL_ differed between laps, being faster in lap 1 than in laps 2 and 3 (*p* < 0.05), with no main effects of age category or performance group. However, L × AC and L × PG interactions were observed, and accordingly, JW and YW had faster SS_REL_ than SW in lap 1 but slower in laps 2 and 3. Regarding performance groups, PG60 was faster than PG30 in lap 1 (*p* < 0.05), with no differences in lap 2 or 3.

**Figure 3:**
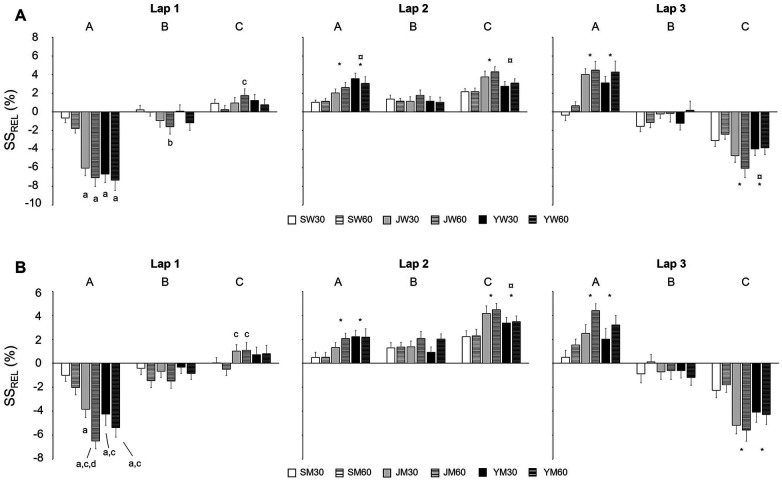
The relative skiing speed (SS_REL_, %) in sections A, B and C during the laps 1, 2 and 3 for women **(A)** and men **(B)** SW30, senior women ranked 1–30; SW60, senior women ranked 31–60; JW30, junior women ranked 1–30; JW60, junior women ranked 31–60; YW30, youth women ranked 1–30; YW60, youth women ranked 31–60; SM30, senior men ranked 1–30; SM60, senior men ranked 31–60; JM30, junior men ranked 1–30; JM60, junior men ranked 31–60; YM30, youth men ranked 1–30; YM60, youth men ranked 31–60. a *p* < 0.05 vs. SW30/SM30 and SW60/SM60; b *p* < 0.05 vs. SW30; c *p* < 0.05 vs. SW60/SM60; d *p* < 0.05 vs. JM30 and YM30 based on significant S × AC × PG interaction effect. * *p* < 0.05 vs. SW/SM, and ¤ *p* < 0.05 vs. JW/JM based on significant S × AC interaction effect without covariates.

The results of repeated-measures ANOVA including F-values, significance levels and effect sizes for SS_REL_ during sections within all three laps are presented in Table 4. According to main effect of section (Figure 3), SS_REL_ differed within each lap. In lap 1, SS_REL_ was fastest in section A and slowest in C (*p* < 0.05). In lap 2, section B was faster than A and C, and section A was also faster than section C (both *p* < 0.05). In lap 3, SS_REL_ was slowest in A and fastest in C (*p* < 0.05). Also, main effects of age category (laps 1–3) and performance group (lap 1) were present.

**Table 4:** Results for repeated-measures ANOVA for relative skiing speed (SS_REL_, %) in women, including *F*- and *p*-values and partial eta-squared effect sizes (*η*^2^_p_) for laps 1, 2 and 3. The upper panel presents analyses without covariates, whereas the lower panel presents analyses including penalty time in prone (PT_PRONE_) and standing (PT_STAND_) as covariates for laps 2 and 3, respectively.

No covariates	Lap 1	Lap 2	Lap 3
S	***F*_1.6,279.2_** **=** **748.4, *p*** **<** **0.001, *η*^2^_p_** **=** **0.81**	***F*_1.8,316.1_** **=** **112.2, *p*** **<** **0.001, *η*^2^_p_** **=** **0.39**	***F*_1.8,317.3_** **=** **792.2, *p*** **<** **0.001, *η*^2^_p_** **=** **0.82**
AC	***F*_2,174_** **=** **38.9, *p*** **<** **0.001, *η*^2^_p_** **=** **0.31**	***F*_2,174_** **=** **23.7, *p*** **<** **0.001, *η*^2^_p_** **=** **0.21**	***F*_2,174_** **=** **8.0, *p*** **<** **0.001, *η*^2^_p_** **=** **0.08**
PG	***F*_1,174_** **=** **7.7, *p*** **<** **0.01, *η*^2^_p_** **=** **0.04**	*F*_1,174_ = 1.2, *p* = 0.268, *η*^2^_p_ = 0.01	*F*_1,174_ = 3.5, *p* = 0.062, *η*^2^_p_ = 0.02
S × AC	***F*_3.2,279.2_** **=** **92.7, *p*** **<** **0.001, *η*^2^_p_** **=** **0.52**	***F*_3.6,316.1_** **=** **26.2, *p*** **<** **0.001, *η*^2^_p_** **=** **0.23**	***F*_3.7,317.3_** **=** **71.4, *p*** **<** **0.001, *η*^2^_p_** **=** **0.45**
S × PG	***F*_1.6,279.2_** **=** **3.7, *p*** **<** **0.05, *η*^2^_p_** **=** **0.02**	*F*_1.8,316.1_ = 0.6, *p* = 0.548, *η*^2^_p_ = 0.00	***F*_1.8,317.3_** **=** **5.6, *p*** **<** **0.01, *η*^2^_p_** **=** **0.03**
S × AC × PG	***F*_3.2,279.2_** **=** **2.6, *p*** **<** **0.05, *η*^2^_p_** **=** **0.03**	*F*_3.6,316.1_ = 1.0, *p* = 0.405, *η*^2^_p_ = 0.01	*F*_3.7,317.3_ = 1.6, *p* = 0.189, *η*^2^_p_ = 0.02

PT_PRONE_ (Lap 2) and PT_STAND_ (Lap 3) as covariates	Lap 1	Lap 2	Lap 3
S	–	***F*_1.8,316.6_** **=** **54.4, *p*** **<** **0.001, *η*^2^_p_** **=** **0.24**	***F*_1.8,314.5_** **=** **236.5, *p*** **<** **0.001, *η*^2^_p_** **=** **0.58**
AC	–	***F*_2,173_** **=** **19.9, *p*** **<** **0.001, *η*^2^_p_** **=** **0.19**	***F*_2,173_** **=** **5.2, *p*** **<** **0.01, *η*^2^_p_** **=** **0.06**
PG	–	*F*_1,173_ = 0.4, *p* = 0.533, *η*^2^_p_ = 0.00	*F*_1,173_ = 1.0, *p* = 0.330, *η*^2^_p_ = 0.01
S × AC	–	***F*_3.7,316.6_** **=** **25.0, *p*** **<** **0.001, *η*^2^_p_** **=** **0.22**	***F*_3.6,314.5_** **=** **68.7, *p*** **<** **0.001, *η*^2^_p_** **=** **0.44**
S × PG	–	*F*_1.8,316.6_ = 1.6, *p* = 0.210, *η*^2^_p_ = 0.01	***F*_1.8,314.5_** **=** **6.3, *p*** **<** **0.01, *η*^2^_p_** **=** **0.04**
S × AC × PG	–	*F*_3.7,316.6_ = 0.8, *p* = 0.521, *η*^2^_p_ = 0.01	*F*_3.6,314.5_ = 1.4, *p* = 0.252, *η*^2^_p_ = 0.02
PT (covariate)	–	*F*_1,173_ = 3.0, *p* = 0.085, *η*^2^_p_ = 0.02	***F*_1,173_** **=** **1.4, *p*** **<** **0.01, *η*^2^_p_** **=** **0.05**
S × PT (covariate)	–	***F*_1.8,316.6_** **=** **4.8, *p*** **<** **0.05, *η*^2^_p_** **=** **0.03**	*F*_1.8,314.5_ = 1.0, *p* = 353, *η*^2^_p_ = 0.01

S, sections; AC, age categories; PG, performance groups.

The bold values indicated statistically significant results.

A significant S × AC × PG interaction was found in lap 1 (Table 4). SW30 and SW60 showed slower SS_REL_ in section A than all JW and YW groups (Figure 3). In section B, JW60 had faster SS_REL_ than SW30, whereas in section C, JW60 had slower SS_REL_ than SW60. Despite a significant S × PG interaction, no pairwise comparisons between performance groups within the same section reached statistical significance.

During lap 2, a significant S × AC interaction was observed (Figure 3). In section A, SW showed faster SS_REL_ than both JW and YW, and JW had faster SS_REL_ than YW. No differences were observed in section B. In section C, both SW and YW had faster SS_REL_ than JW. Including PT_PRONE_ as a covariate resulted in a significant interaction effect (Table 4) but however, the significant pairwise comparisons (S × AC) remained similar as without inclusion of covariate.

During lap 3, a significant S × AC interaction was also observed. SW showed faster SS_REL_ than both JW and YW in section A, whereas no differences were found in section B (Figure 3). In section C, both JW and YW had faster SS_REL_ than SW, and JW also had faster SS_REL_ than YM. Despite a significant S × PG interaction effect, no pairwise comparisons between performance groups within the same section were significant. Including PT_STAND_ as a covariate resulted in a main effect (Table 4). However, the significant pairwise comparisons according to S × AC interaction effect remained unchanged following the inclusion of the covariate.

### 3.2 Between and within laps pacing for men

The repeated-measures ANOVA results including F-values, significance levels and effect sizes for SS_REL_ for men during all three laps are presented in Figure 2. SS_REL_ differed across laps, age categories, and performance groups. SS_REL_ was faster in lap 1 than in laps 2 and 3 (both *p* < 0.05). JM differed from both SM and YM (both *p* < 0.05), and differences were also observed between PG30 and PG60 (*p* < 0.05). The L × AC interaction showed that JM had faster SS_REL_ than both SM and YM in lap 1. YM also had faster SS_REL_ than SM in lap 1. In lap 2 both JM and YM had slower SS_REL_ than SM. In lap 3, JM had slower SS_REL_ than both SM and YM. For the L × PG interaction, PG60 had faster SS_REL_ than PG30 in lap 1 but slower in lap 3 (both *p* < 0.05).

The results of repeated-measures ANOVA, including F-values, significance levels, and effect sizes for SS_REL_ during sections within all three laps are presented in Table 5. SS_REL_ differed across sections within each lap (Figure 3). In lap 1, SS_REL_ was fastest in section A and slowest in section C (all *p* < 0.05). In lap 2, SS_REL_ was faster in sections A and B than in section C (both *p* < 0.05), whereas in lap 3, SS_REL_ was slowest in section A and fastest in section C (all *p* < 0.05).

**Table 5:** Results for repeated-measures ANOVA for relative skiing speed (SS_REL_, %) in men, including *F*- and *p*-values and partial eta-squared effect sizes (*η*^2^_p_) for laps 1, 2 and 3. The upper panel presents analyses without covariates, whereas the lower panel presents analyses including penalty time in prone (PT_PRONE_) and standing (PT_STAND_) as covariates for laps 2 and 3, respectively.

No covariates	Lap 1	Lap 2	Lap 3
S	***F*_1.6,287.4_** **=** **485.9.4, *p*** **<** **0.001, *η*^2^_p_** **=** **0.74**	***F*_1.9.329.9_** **=** **147.7, *p*** **<** **0.001, *η*^2^_p_** **=** **0.46**	***F*_1.7,296.2_** **=** **814.6, *p*** **<** **0.001, *η*^2^_p_** **=** **0.82**
AC	***F*_2,174_** **=** **7.2, *p*** **<** **0.001, *η*^2^_p_** **=** **0.08**	***F*_2,174_** **=** **30.0, *p*** **<** **0.001, *η*^2^_p_** **=** **0.26**	*F*_2,174_ = 1.3, *p* = 0.283, *η*^2^_p_ = 0.01
PG	***F*_1,174_** **=** **20.0, *p*** **<** **0.001, *η*^2^_p_** **=** **0.10**	***F*_1,174_** **=** **6.3, *p*** **<** **0.05, *η*^2^_p_** **=** **0.04**	***F*_1,174_** **=** **5.1, *p*** **<** **0.05, *η*^2^_p_** **=** **0.03**
S × AC	***F*_3.3,287.4_** **=** **65.4, *p*** **<** **0.001, *η*^2^_p_** **=** **0.43**	***F*_3.8,329.9_** **=** **13.0, *p*** **<** **0.001, *η*^2^_p_** **=** **0.13**	***F*_3.4,296.2_** **=** **60.9, *p*** **<** **0.001, *η*^2^_p_** **=** **0.42**
S × PG	***F*_1.7,287.42_** **=** **13.6, *p*** **<** **0.001, *η*^2^_p_** **=** **0.07**	*F*_1.9,329.9_ = 2.0, *p* = 0.139, *η*^2^_p_ = 0.01	***F*_1.7,296.2_** **=** **12.2, *p*** **<** **0.001, *η*^2^_p_** **=** **0.07**
S × AC × PG	***F*_3.3,287.4_** **=** **3.1, *p*** **<** **0.05, *η*^2^_p_** **=** **0.03**	*F*_3.8,329.9_ = 1.5, *p* = 0.216, *η*^2^_p_ = 0.02	*F*_3.4,296.2_ = 2.4, *p* = 0.059, *η*^2^_p_ = 0.03

PT_PRONE_ (Lap 2) and PT_STAND_ (Lap 3) as covariates	Lap 1	Lap 2	Lap 3
S	–	***F*_1.9,327.7_** **=** **60.5, *p*** **<** **0.001, *η*^2^_p_** **=** **0.26**	***F*_1.7,294.7_** **=** **211.3, *p*** **<** **0.001, *η*^2^_p_** **=** **0.55**
AC	–	***F*_2,173_** **=** **22.1, *p*** **<** **0.001, *η*^2^_p_** **=** **0.20**	*F*_2,173_ = 2.0, *p* = 0.134, *η*^2^_p_ = 0.02
PG	–	*F*_1,173_ = 3.5, *p* = 0.063, *η*^2^_p_ = 0.02	*F*_1,173_ = 1.4, *p* = 0.232, *η*^2^_p_ = 0.01
S × AC	–	***F*_3.8,327.7_** **=** **9.7, *p*** **<** **0.001, *η*^2^_p_** **=** **0.10**	***F*_3.4,294.7_** **=** **58.8, *p*** **<** **0.001, *η*^2^_p_** **=** **0.41**
S × PG	–	***F*_1.9,327.7_** **=** **3.3, *p*** **<** **0.05, *η*^2^_p_** **=** **0.02**	***F*_1.7,294.7_** **=** **9.3, *p*** **<** **0.001, *η*^2^_p_** **=** **0.05**
S × AC × PG	–	*F*_3.8,327.7_ = 1.8, *p* = 0.141, *η*^2^_p_ = 0.02	*F*_3.4,294.7_ = 2.3, *p* = 0.069, *η*^2^_p_ = 0.03
PT (covariate)	–	***F*_1,173_** **=** **4.8, *p*** **<** **0.05, *η*^2^_p_** **=** **0.03**	***F*_1,173_** **=** **6.9, *p*** **<** **0.05, *η*^2^_p_** **=** **0.04**
S × PT (covariate)	–	***F*_1.9,327.7_** **=** **4.6, *p*** **<** **0.05, *η*^2^_p_** **=** **0.03**	*F*_1.7,294.7_ = 0.5, *p* = 0.596, *η*^2^_p_ = 0.00

S, sections; AC, age categories; PG, performance groups.

The bold values indicated statistically significant results.

A significant S × AC × PG interaction was observed during lap 1. In section A, SM30 and SM60 showed slower SS_REL_ than all JM and YM groups. In addition, SM60 had slower SS_REL_ than JM60, YM30 and YM60, whereas JM30 and YM30 had slower SS_REL_ than JM60. No group differences were observed in section B. In section C, both JM30 and JM60 had slower SS_REL_ than SM60. According to S × PG interaction, PG60 had faster SS_REL_ than PG30 in sections A and B.

During lap 2, a significant S × AC interaction showed that SM had faster SS_REL_ than JM and YM in sections A and C, while YM had faster SS_REL_ than JM in section C. Including PT_PRONE_ as a covariate resulted in significant main and interaction effects (Table 5), eliminating the main effect of PG and introducing an S × PG interaction in which PG30 showed faster SS_REL_ than PG60 in section B (*p* < 0.05). However, the significant pairwise comparisons according to S × AC interaction effect remained unchanged following the inclusion of the covariate.

During lap 3, a significant S × AC interaction showed that SM had faster SS_REL_ in section A but slower SS_REL_ in section C than JM and YM. Including PT_STAND_ as a covariate resulted in a significant main effect of the covariate (Table 5), eliminating the main effect of PG but not affecting the pairwise comparisons for S × AC interaction. Lastly, according to significant S × PG interaction effect (with or without covariate), PG30 had faster SS_REL_ than PG60 in section A (*p* < 0.05).

### 3.3 Shooting performance

Outcomes of the shooting-related variables are presented in Table 6. In women, SW30 had lower MT_PRONE_ than JW60 and YW60. JW30 also had lower MT_PRONE_ than JW60. In addition, SW30 had lower MT_STAND_ than all other groups except YW30. Regarding penalty time, SW30 showed shorter PT_PRONE_ compared to JW60, YW30 and YW60. SW60 and JW30 also showed shorter PT_PRONE_ compared to JW60 and YW60. For standing shooting, SW30 had shorter PT_STAND_ compared to all other groups. Additionally, YW30 had shorter PT_STAND_ than JW60. Moreover, SW60 and YW30 had shorter PT_STAND_ than YW60.

**Table 6:** Number of missed targets (MT) and penalty times (PT) (median [IQR]) in prone (PRONE) and standing (STAND) shooting positions across the six performance groups in women and men.

Shooting variables	SW30	SW60	JW30	JW60	YW30	YW60	Kruskal–Wallis ANOVA, *p*-value, effect size
MT_PRONE_	0 [1]	1 [1]	0 [1]	1 [2]^A,C^	1 [1]	1 [1]^A^	*χ*^2^ *=* *19.0, p* *<* *0.01, ε^2^* *=* *0.11*
MT_STAND_	0 [1]	1 [1]^A^	1 [1]^A^	1 [0]^A^	1 [1]	1 [1]^A^	*χ*^2^ *=* *27.1, p* *<* *0.001, ε^2^* *=* *0.15*
PT_PRONE_ (sec)	6.7 [24.7]	30.1 [26.5]	7.2 [27.1]	35.8 [43.5]^A,B,C^	32.9 [27.1]^A^	35.7 [30.5]^A,B,C^	*χ*^2^ *=* *49.4, p* *<* *0.001, ε^2^* *=* *0.28*
PT_STAND_ (sec)	5.9 [25.0]	31.8 [26.6]^A^	33.3 [20.3]^A^	36.0 [4.2]^A^	32.8 [28.1]^A,D^	37.5 [32.1]^A,B,E^	*χ*^2^ *=* *56.5, p* *<* *0.001, ε^2^* *=* *0.32*

	SM30	SM60	JM30	JM60	YM30	YM60	Kruskal–Wallis ANOVA, *p*-value, effect size
MT_PRONE_	0 [0]	1 [1]^A^	1 [1]^A^	1 [1]^A^	1 [2]^A^	1 [1]^A,B^	*χ*^2^ *=* *56.4, p* *<* *0.001, ε^2^* *=* *0.32*
MT_STAND_	1 [1]	1 [2]	1 [1]	1 [1]	1 [1]	2 [1]^A,B,C,E^	*χ*^2^ *=* *56.4, p* *<* *0.001, ε^2^* *=* *0.32*
PT_PRONE_ (sec)	6.0 [0.9]	28.1 [23.9]^A^	30.9 [24.8]^A^	32.6 [28.1]^A,B^	31.0 [40.1]^A^	32.2 [27.2]^A,B^	*χ*^2^ *=* *56.4, p* *<* *0.001, ε^2^* *=* *0.32*
PT_STAND_ (sec)	27.1 [24.3]	29.6 [40.8]	30.5 [26.5]	34.8 [30.1]^A,C^	29.8 [25.2]^D^	55.3 [27.5]^A,B,C,E^	*χ*^2^ *=* *43.7, p* *<* *0.001, ε^2^* *=* *0.24*

SW30, senior women ranked 1–30; SW60, senior women ranked 31–60; JW30, junior women ranked 1–30; JW60, junior women ranked 31–60; YW30, youth women ranked 1–30; YW60, youth women ranked 31–60; SM30, senior men ranked 1–30; SM60, senior men ranked 31–60; JM30, junior men ranked 1–30; JM60, junior men ranked 31–60; YM30, youth men ranked 1–30; YM60, youth men ranked 31–60). *χ*^2^ and *ε^2^* values refer to Kruskal–Wallis ANOVA and epsilon squared effect size.

A vs. SW30/SM30, ^B^ vs. SW60/SM30, ^C^ vs. JW30/JM30, ^D^ vs. JW60/JM60, ^E^ vs. YW30/YM30, all *p* < 0.05.

In men, SM30 had lower MT_PRONE_ than all other groups. For standing shooting, all groups had similar MT_STAND_ except YM60. Regarding penalty time, SM30 had shorter PT_PRONE_ than all other groups. Furthermore, SM60 had shorter PT_PRONE_ than JM60 and YM60. For standing shooting, SM30 had shorter PT_STAND_ than JM60. In contrast, YM60 had longer PT_STAND_ than all other groups except JM60. In addition, JM60 had longer PT_STAND_ than JM30 and YM30.

## 4 Discussion

The present study compared pacing patterns among elite international level biathletes across the senior, junior, and youth age categories during a biathlon sprint competition while accounting for performance level. The main findings regarding lap pacing were that (1) SS_REL_ was faster during lap 1 than during laps 2 and 3 in both women and men, (2) senior biathletes demonstrated more even pacing in terms of SS_REL_ than junior and youth biathletes across all laps, and (3) higher-performing biathletes (PG30), particularly among men, demonstrated more even pacing compared with lower-performing biathletes (PG60). Accordingly, these finding partly confirm the preset hypothesis. Regarding sectional SS_REL_, the results further indicated that (4) differences between age categories were primarily observed in skiing sections A and C, (5) differences between performance groups in men were present in section A but also in section B, and finally (6) preceding shooting performance in terms of penalty time, resulted in significant main and/or interaction effects on SS_REL_ during the subsequent skiing segment without influencing the pairwise comparisons.

### 4.1 Pacing between and within laps

The present findings partly support the typical J-shaped pacing pattern observed in biathlon, characterized by the fastest first lap, followed by a slower middle lap and a slightly faster final lap (2, 4, 13). In the present study, lap 1 was fastest whereas no difference was observed between laps 2 and 3. The reason for this discrepancy between studies is unknown but may be linked to the number of competitions included in the data analysis. The present study used data only from one competition, whereas the previous biathlon studies have used larger datasets averaging data from several competitions (2, 4). However, when comparing pacing patterns across the three age categories (senior, junior, and youth), senior biathletes demonstrated more even pacing across the three laps. For example, during lap 1, the junior and youth groups skied approximately 2.3–3.1 (women) and 1.2–2.2 (men) percentage points faster (SS_REL_) than senior women and men, respectively (Figure 2). In contrast, during lap 2, senior women and men showed 0.7–0.9 and 1.0–1.0 percentage points faster SS_REL_, respectively, than the junior and youth groups within the same sex. A similar pattern was observed during the lap 3, where senior women and men were 1.8–2.2 and 0.1–1.2 percentage points faster than junior and youth groups. This observation of a faster initial phase among younger biathletes aligns well with previous research in cross-country skiing (25, 28, 29).

Consistent with previous findings in biathlon (2–4, 13), and similar to observations in cross-country skiing (20, 21, 30), the present results also indicate that the highest-performing biathletes (PG30) adopt a more even pacing strategy than lower-ranked biathletes (PG60), despite overall performance depending on both skiing and shooting components in biathlon. In both sexes, PG60 was faster during lap 1 with no difference between performance groups during lap 2. During lap 3, PG30 was faster than PG60, but only in men.

When examining sectional performance within laps, the present findings indicate that skiing speed in sections A and C was the primary determinant of r the overall SS_REL_ during each lap. For example, during lap 1, the differences between age categories were primarily explained by faster SS_REL_ in section A among junior and youth groups compared with seniors. In contrast, during lap 2, when senior groups were faster than the junior and youth groups, the differences in SS_REL_ were attributable to faster SS_REL_ in both sections A and C among the seniors. During lap 3, seniors were faster in section A but slower in section C. Since section C constituted a smaller proportion of the lap (18 or 29% of total lap length) than section A, its contribution to the overall SS_REL_ for the entire lap was therefore smaller. For section A, it should be noted that its relative length differed between youth and both senior and junior groups (women 33% vs. 44% and men 44% vs. 58% of the total lap length, respectively), which may have contributed to differences in overall SS_REL_. However, senior and junior groups skied the same course, with identical section lengths and topography, yet displayed different pacing patterns. This suggests that the more even pacing pattern observed among senior biathletes is largely explained by differences in SS_REL_ during the initial phase of each lap (section A), rather than differences in course characteristics.

According to the present results, only a few differences were observed between the junior and youth groups in both sexes: during lap 2 in section A, JW had faster SS_REL_ than YW, while in section C, YW and YM were faster than JW and JM, respectively. During lap 3, JW was faster than YW in section C. Therefore, the few differences observed between the two younger age categories suggest that, when considering pacing patterns across all three laps, it is more meaningful to focus on the differences between the combined junior-youth groups and the senior groups.

The reasons underlying the differences in pacing strategies between junior-youth biathletes and seniors are unclear, but they may relate to a combination of maturation and accumulated experience as suggested in litterature. During adolescence, the ability to draw on previous experiences to regulate pacing behaviour is still developing (31), and as pacing is considered as a self-regulatory skill, relying on the ability to plan, monitor, and reflect on goal-directed processes (32, 33), age- and experience-related differences in these capacities may partly explain the observed pacing patterns in the present study. Consequently, greater task-specific experience (e.g., seniors) may enable biathletes to more consistently achieve a predetermined pace, resulting in pacing behaviour that more closely reflects the demands of the task (34). However, it is important to point out that these aspects have not been studied in the present study. Nevertheless, since highly trained biathletes exhibiting a pronounced fast-start strategy are shown to improve skiing performance by adopting a more even pacing profile (13), biathletes and coaches may benefit from monitoring pacing behaviour during training and competition, as previously suggested in other sports (35).

Another interesting finding was that during lap 3, both the junior and youth groups demonstrated faster SS_REL_ in section C than senior groups when approaching the finish line. This contrasts with laps 1 and 2, when the same section led to the shooting range and biathletes intentionally decrease the exercise intensity in preparation to shooting (15). During lap 3, junior women and men, as well as youth women and men, showed slightly larger increases in absolute skiing speed in section C (juniors + 0.6 m·s^−1^, youth + 0.5 m·s^−1^; data not shown), than both senior women and men (+0.4 m·s^−1^) compared to their average skiing speeds in the section C during laps 1 and 2. One possible explanation is that junior and youth biathletes reduce their skiing speed to a greater extent in section C during laps 1 and 2 in preparation for shooting, which in turn allows them to ski faster in this section when approaching the finish. Conversely, senior biathletes may approach the shooting range with less reduction in skiing speed than younger biathletes. Consequently, they may have a reduced capacity to further increase their speed in the final approach (section C) to the finish line, possibly because they have expended greater effort in earlier during Lap 3 (section A), thereby limiting their ability to further increase skiing speed.

### 4.2 Effect of shooting performance on pacing

As penalty loops resulting from missed targets have been suggested to alter the pacing profiles in biathlon (1, 36), the present study also considered the possible effect of shooting performance on skiing performance during the subsequent skiing lap. Therefore, penalty time, defined as the time spent skiing from the end of the shooting range to the first timing point where the skiing laps begun, including any penalty loop(s) resulting from missed target(s), was used as a covariate in the additional repeated-measures ANOVA of SS_REL_ for laps 2 and 3.

For both women and men, including PT_PRONE_ as a covariate in the sectional analysis of lap 2 revealed a significant interaction effect (also main effect for men). This suggest that the association between PT_PRONE_ and SS_REL_ varied across sections of the lap, indicating that changes in SS_REL_ may, in part, be related to biathletes’ PT_PRONE_. Similarly, including PT_STAND_ as a covariate in sectional analysis of lap 3 revealed a significant main effect for both women and men, suggesting that PT_STAND_ was associated with SS_REL_ following standing shooting bout. However, the inclusion of these covariates did not alter the significant pairwise comparisons identified in the repeated-measures ANOVA without covariates.

The influence of PT_PRONE_ and PT_STAND_ as covariates should, however, be interpreted with caution. Analyses of the relationships between penalty times (PT_PRONE_ and PT_STAND_) and subsequent SS_REL_ in section A during laps 2 and 3 revealed significant correlations across all groups (all groups combined; data not shown), although both PT_PRONE_ and PT_STAND_ were clustered according to the number of missed targets. Altogether, these findings suggests that the additional skiing required for penalty loops (150 m per missed shot, ∼ 25 s) may induce fatigue, potentially impairing performance during subsequent lap or section, as reflected in the observed covariate effects. At the same time, penalty time itself includes a skiing component and may therefore also reflect the biathlete's overall skiing capacity. Thus, faster skiers are likely to complete penalty loops more quickly, regardless of the number of missed targets. Consequently, this potential confounding factor should be considered more carefully in the future research.

### 4.3 Strengths and limitations

A primary strength of the present study was that the cohort included the best biathletes from three age categories competing at the highest international level in biathlon sprint competitions (IBU Youth/Junior World Championships and the IBU World Cup). The results of these sprint competitions also determined the start lists for the subsequent pursuit competitions, in which the 60 highest-ranked biathletes from the sprint qualified to compete. Therefore, all biathletes were likely to have performed with maximal effort during the analysed sprint competitions: athletes in the PG30 groups competed for podium finishes or top 10 positions, while those in the PG60 groups competed for a place in the subsequent pursuit competition.

A second strength of the study was that the weather conditions were comparable across competitions. For example, wind speed was similar across events, ranging from 0.6 to 2.0 m·s⁻^1^, and therefore likely had a minimal external influence on skiing performance. Second, the air temperature ranged from −0.7 to 3.7 °C and especially, snow temperatures, ranging from −0.7 to 0.0 °C, were comparable between competitions. Third, the skiing conditions were consistently hard-packed during all competitions. However, although the organiser prepared the courses for both competition seasons using machine-made snow, which is generally less variable in structure and provides a harder skiing surface than natural snow (37), potential differences in snow properties between events, and their possible influence on skiing performance, cannot be entirely excluded.

Third, both events (the IBU World Cup and the Youth/Junior World Championships) were conducted on identical skiing courses, with identically placed intermediate timing points, thereby enabling sectional analyses between competitions. The only difference was a 25 m discrepancy in the length of section C during lap 3 between senior and youth/junior events. However, the course topography also represents one limitation of the present study. Senior and junior men skied three laps of 3.3 km, in which section A was ∼ 800 m longer than the corresponding section for youth men. This section also included an uphill from the lowest to the highest point of the course, with a maximal climb of 42 m compared with 18 m for youth men. Thus, this may have influenced pacing among youth men although they exhibited a pacing pattern similar to that of junior men, who skied the same course as senior men.

The main limitation of the study was that the present results were based on competitions conducted only at a single venue with specific course topography. Therefore, the future studies should examine pacing strategies among these age categories across different venues and course profiles to verify the present findings. Such studies could also investigate whether different topographical approaches to the shooting range influence shooting performance. Another limitation is that the Youth/Junior World Championships were held at the end of the 2024/2025 competition season (March 2025), whereas the IBU World Cup took place at the beginning of the following season (November 2025), resulting in an approximately eight-month interval between the events. Although skiing conditions likely were comparable between the competitions, potential seasonal differences in athletes' training status and competition readiness cannot be excluded.

## 5 Conclusions

The present findings demonstrate, for the first time and in an ecological setting, differences in pacing strategies between senior, junior, and youth biathletes. Senior biathletes exhibited more even pacing than junior and youth biathletes across all three laps and within the different sections of each lap. This more even pacing pattern was also observed among higher-performing biathletes compared with lower-performing biathletes in men. However, these findings should be interpreted with some caution, as section lengths within the lap differed between the youth groups and both the senior and junior groups. Furthermore, preceding shooting performance may influence subsequent skiing performance; however, this relationship requires further investigation. Based on the present findings, biathletes and their coaches are encouraged to monitor pacing behaviour during training and competition to increase awareness of pacing strategies and to reflect on the experience gained.

## Data Availability

Publicly available datasets were analyzed in this study. This data can be found here: https://www.biathlonresults.com/#/datacenter.
